# Artificial Intelligence in Adult and Pediatric Dentistry: A Narrative Review

**DOI:** 10.3390/bioengineering11050431

**Published:** 2024-04-27

**Authors:** Seyed Mohammadrasoul Naeimi, Shayan Darvish, Bahareh Nazemi Salman, Ionut Luchian

**Affiliations:** 1 School of Dentistry, Zanjan University of Medical Sciences, Zanjan 4513956184, Iran; emadnaeiminasir@gmail.com; 2 School of Dentistry, University of Michigan, Ann Arbor, MI 48104, USA; sdarvish@umich.edu; 3 Department of Pediatric Dentistry, School of Dentistry, Zanjan University of Medical Sciences, Zanjan 4513956184, Iran; 4 Department of Periodontology, Faculty of Dental Medicine, “Grigore T. Popa” University of Medicine and Pharmacy, 700115 Iasi, Romania

**Keywords:** artificial intelligence, machine learning, deep learning, pediatrics, dentistry

## Abstract

Artificial intelligence (AI) has been recently introduced into clinical dentistry, and it has assisted professionals in analyzing medical data with unprecedented speed and an accuracy level comparable to humans. With the help of AI, meaningful information can be extracted from dental databases, especially dental radiographs, to devise machine learning (a subset of AI) models. This study focuses on models that can diagnose and assist with clinical conditions such as oral cancers, early childhood caries, deciduous teeth numbering, periodontal bone loss, cysts, peri-implantitis, osteoporosis, locating minor apical foramen, orthodontic landmark identification, temporomandibular joint disorders, and more. The aim of the authors was to outline by means of a review the state-of-the-art applications of AI technologies in several dental subfields and to discuss the efficacy of machine learning algorithms, especially convolutional neural networks (CNNs), among different types of patients, such as pediatric cases, that were neglected by previous reviews. They performed an electronic search in PubMed, Google Scholar, Scopus, and Medline to locate relevant articles. They concluded that even though clinicians encounter challenges in implementing AI technologies, such as data management, limited processing capabilities, and biased outcomes, they have observed positive results, such as decreased diagnosis costs and time, as well as early cancer detection. Thus, further research and development should be considered to address the existing complications.

## 1. Introduction

Information technology has experienced significant advancements in recent years, leading to a dramatic surge in the volume of data. The need for processing large datasets, also known as “big data”, has led to an increasing demand for the application of artificial intelligence [1]. The term “artificial intelligence” (AI) was initially introduced by McCarthy in the 1950s and implied the concept of developing machines that can perform tasks typically carried out by humans [2,3]. AI refers to the ability of machines to simulate human intelligence and undertake intricate tasks like problem-solving, decision-making, and recognition of objects [4]. As a subset of AI, “machine learning” (ML) is expected to make remarkable contributions to clinical diagnosis due to its reasonable predictive accuracy. In addition, ML models can process data with diverse characteristics, which is a task beyond the scope of conventional analysis methods [5]. The connection between AI and medicine dates back to the 1970s when projects such as the Stanford University experimental computer for AI in medicine and the early-stage expert systems were launched [6]. Neural networks represent a specific category of machine learning algorithms. The network imitates the structure of the human brain by forming a computational network of cells arranged in a series of layers. “Deep learning” (DL) refers to multi-layered neural networks [7]. By improving performance on complex tasks in areas like image processing (e.g., object detection and facial identification) and sound processing, DL has taken human performance to new heights [8]. ML algorithms have facilitated clinical decision-making by aiding in the prognosis and diagnosis of various health conditions, representing the main areas of progress for AI in the medical field [4]. AI has found numerous applications in the medical field, ranging from radiology, dermatology, and neurology to ophthalmology, oncology, cardiology, genetics, emergency medicine, and drug design [9]. Other significant areas of healthcare, such as dentistry, have also adopted ML techniques. The integration of digitized imaging and electronic medical records in dental practice enables the implementation of virtual AI algorithms like support vector machine (SVM), artificial neural network (ANN), and especially convolutional neural network (CNN) [10]. CNNs are the preferred choice for image classification due to their ability to automatically extract features through repeated convolution and pooling. This unique architecture, with multiple layers containing learnable filters, enables CNNs to excel in medical computer vision tasks, making them the preferred option for AI-driven computer vision applications in dental practice, which accounts for the majority of research conducted on AI in dentistry [11,12]. 

AI has been employed to identify and detect different variables from dental radiographs. Different measures are used to determine the overall diagnostic performance of AI in dental science, such as accuracy (the ratio of correctly classified samples to all samples), sensitivity (the probability of getting a positive outcome when the condition is present), and specificity (the probability of getting a negative outcome when the condition is not present). The reporting of a metric depends on the objectives and methodologies employed by researchers [13]. There have been several research studies examining the implementation of machine learning in dentistry, including the prediction of postoperative pain [14], periodontal bone loss measurement [15], the need for tooth extraction in orthodontic treatment [16], and the presence of root caries [17]. Recently, some reviews have been published about the implementation of AI in specific subfields of dentistry, such as orthodontics [18], radiology [7], and endodontics [19]. However, only a few studies have approached the application of AI in the broad spectrum of dental subfields, and there is a lack of comprehensive reviews covering different aspects of dental science at once. Children, unlike adults, tend to be more uncooperative during dental procedures, and managing child patients is typically more challenging than managing adults [20]. This poses challenges when repeating tasks such as dental radiographs. However, AI can aid practitioners in diagnosis, potentially minimizing the need for repeated procedures. Moreover, health organizations have advised routine dental screenings for children to prevent the development of dental problems like caries, which may not show symptoms in the initial stages [21]. Utilizing the computational capabilities of AI software, these screenings can be enhanced, and early detection of oral conditions can be facilitated. This study not only covers the general applications of AI in dentistry but also includes some of the recent implementations of AI in pediatric patients.

Considering the potential of AI for reducing costs and errors, decision-making, and diagnosis, this study aims to summarize AI-based technologies in various dental subfields and provide insight into this rapidly advancing healthcare domain (Table 1). 

## 2. Materials and Methods

A comprehensive electronic search was conducted using MeSH terms, including artificial intelligence, machine learning, deep learning, pediatrics, and dentistry, from the inception of available data until February 2024 across online databases such as PubMed, Google Scholar, Scopus, and Medline. Additionally, non-MeSH terms such as implantology, cariology, VRF, and odontogenic were manually searched in combination with the aforementioned MeSH terms using Boolean operators in order to ensure the inclusion of appropriate papers. Papers with higher citation rates and those published in reputable journals within their respective fields were prioritized, aiming to include the most credible AI applications in dental practice. Non-English articles and incomplete papers were excluded. Preference was given to papers that emphasized the clinical application of AI in dentistry rather than focusing solely on theoretical aspects of AI algorithms. Ultimately, 84 papers were chosen as the most relevant for the purposes of this review.

## 3. Review

AI in dentistry excels at detecting bone loss and predicting pain in periodontics, diagnosing conditions like cleft palate in oral and maxillofacial surgery, designing dental arches in prosthodontics, and detecting implant systems and complications in implantology. It also aids in treatment planning in orthodontics and predicts plaque and treatment needs in pediatric dentistry and cariology. In the upcoming sections, we will explore AI applications in each field individually.

### 3.1. Applications of AI in the Field of Periodontics

Periodontal disease is a multifactorial condition that results from the inflammatory response of the host’s immune system to multiple bacterial species in the oral cavity [22]. Periodontitis ranks as the sixth most widespread disease globally, and it can cause periodontal bone loss (PBL). Hence, early detection of PBL, which can be a complex task for novice practitioners, is crucial for the diagnosis and treatment of periodontitis [53]. Using a deep neural network on periapical radiographs from 63 patients, Danks et al. estimated the PBL and its severity with a combined accuracy of 83.3% [15]. 

Kim et al. used ML to estimate the level of periodontitis progression based on salivary samples. They collected mouthwash samples from 692 patients and measured the copy number of the nine most important bacterial species that cause periodontal disease. The input features for the ML algorithms (NN, RF, SVM, RLR) were the copy numbers of the pathogens [22]. Two studies compared different ML models to find the best-performing algorithm for detecting the most important factors in periodontal disease. They both demonstrated that the decision-tree model can identify the complex risk factors for PD with higher accuracy and sensitivity than other models [23,54]. 

AI can take multiple factors of periodontal disease into account for diagnosis with high accuracy, which can be a challenging and arduous task for dental practitioners. Therefore, with the help of ML, dentists can determine periodontitis sooner than before, prevent further complications, and decrease treatment costs for the patient.

### 3.2. Applications of AI in the Field of Endodontics

AI has been employed in endodontics to detect fractures and several other tooth characteristics, such as minor apical foramen, to increase successful treatment outcomes [55]. Root canal treatment (RCT) can result in postoperative pain, which is affected by various factors, and anticipating the pain is crucial for the planning of subsequent therapeutic schedules. Xin Gao et al. used an ANN model to predict postoperative pain after receiving RCT. The AI model was trained using input data such as medication during RCT, oral hygiene, gender, and age. The perceived pain was graded according to the patient’s subjective experience, and the final accuracy of the model was 95.60% [14]. Several studies have implemented ML methods to locate minor apical foramen and calculate working length. While placing the root filling material, instrumentation beyond the apical foramen should be avoided. Accurate measurement of the working length (WL) and limiting the placement of root-filling material only to the canal can prevent exacerbation of postoperative pain and reduce the periapical immune response to RCT material [55]. Saghiri et al. compared the accuracy of an ANN in finding the location of the minor apical foramen to the assessment done by endodontists (which was accurate in 76% of the teeth). Surprisingly, AI yielded a higher accuracy of 96% [24,55]. Vertical root fractures (VRFs) are a significant complication, accounting for 2% to 5% of crown/root fractures, and they may require either root resection or tooth extraction [19]. With early detection of VRFs, dentists can save other remaining roots. Fukuda et al. used a convolutional neural network (CNN) to detect VRFs with a precision of 93% on three hundred panoramic radiographs, which included a total of 330 VRF teeth with clearly discernible fractures [25]. Moreover, recognizing the shape of the root canal can be extremely helpful in multiple endodontic treatments. Benyo et al. detected the medial axis of the root canal based on the fuzzy clustering method in order to provide a better understanding of the root morphology [26]. Molars have always been of great importance to endodontists. AI was utilized for the classification of mandibular third molars [27], prediction of third molar eruption [28], and recognition of molar root morphology [29]. 

In endodontics, AI contributes to treatment when it comes to detecting minute tooth characteristics’ alterations that can be hard to recognize for humans.

### 3.3. Applications of AI in the Field of Oral and Maxillofacial Surgery

ML models have performed well in various surgical procedures, including orthognathic surgery, landmark detection, cyst and lesion detection, and osteoporosis classification. Orthognathic treatment aims to address both functional and aesthetic issues related to dentofacial deformities by utilizing a combination of orthodontic and surgical interventions. Research studies have been conducted on assessing the need for orthognathic surgery, evaluation of maxillary sinusitis, diagnosis of orthognathic surgery, and blood loss during surgery. Patcas et al. applied AI to evaluate the influence of orthognathic treatment on facial esthetics and estimated age. According to this study, 66.4% of the patients had improved appearance after the surgery [30].

Cleft palate (CP) and cleft alveolus (CA) are congenital conditions that create challenges for children and their families. Early surgical interventions to stabilize maxillary segments are typically done around ages 8–10. Panoramic radiography plays a vital role in evaluating cleft status. However, interpreting these images can be challenging for inexperienced radiologists. Kuwada et al. used panoramic radiographs from 383 children to create a DL model capable of identifying cleft alveolus (CA) and cleft palate (CP) that can assist clinicians in detecting clefts [31]. 

Kwon et al. developed a CNN model for automatic diagnosis of odontogenic cysts and tumors in the upper and lower jaws using panoramic radiographs. The CNN model was trained on both histopathological diagnoses from biopsies and clinical diagnosis by two radiologists with over 15 years of experience, resulting in a 95.6% accuracy rate in the classification of lesions [33]. Osteoporosis is a systemic skeletal disorder distinguished by a reduction in bone density and deterioration of bone architecture, leading to a higher risk of bone fractures and pain [56]. Several studies have used dental radiographs to classify mandibular bone density for early detection of osteoporosis. Alzubaidi et al. calculated the thickness and roughness of the mandibular cortical bone and employed a support vector machine (SVM) to categorize each Dental Panoramic Radiograph (DPR) into three groups based on the probability of osteoporosis [32]. The gold standard for the detection of oral lesions and diseases is based on histopathology, which can be invasive and time-consuming. In contrast, diagnosis with high-precision AI models is non-invasive and can vastly reduce treatment costs. Moreover, as Immanuel Kant has stated, aesthetic judgment is a subjective task and is prone to human error. Therefore, AI can help us achieve a universal standard for the assessment of post-surgery appearance without human bias.

### 3.4. Applications of AI in the Field of Prosthodontics

In prosthodontics, AI technology is still scarcely used. AI has been applied to computer-aided design and manufacturing systems, removable partial dentures, implant prosthetics, and orofacial anatomy [57]. Designing removable partial dentures (RPDs) is based on the classification of the dental arch as the first step. Takahashi et al. utilized a CNN to classify dental arches. A total of 1184 dental arch images were employed, with 748 images for the maxilla and 436 images for the mandible. The images were categorized into four types of dental arches: edentulous, complete dentition, arches with missing posterior teeth, and arches with limited edentulous space. The classification accuracy of the model was found to be 99.5% for the maxilla and 99.7% for the mandible [34]. 

To effectively plan dental treatments for patients, obtaining detailed information about the condition of their teeth and mouth is essential. A recent study conducted in 2021 utilized a DL approach to develop a method for identifying dental prostheses and restorations by analyzing 1904 oral photographic images of dental arches. This approach aimed to improve the accuracy and quality of intraoral data available for treatment planning purposes [35].

### 3.5. Applications of AI in the Field of Dental Implantology 

For over half a century, dental implants have been utilized as a dependable long-term solution (with a success rate of over 90% lasting for more than ten years) with the purpose of replacing missing teeth [36]. However, mechanical and biological complications, such as screw loosening, fractures, low stability, and peri-implantitis, occur frequently [58].

Dental implant systems come in a wide variety, with over 4000 different types produced by several companies. Additionally, there is a variety of fixture structures available, including straight, tapered, conical, elliptical, trapezoidal, internal, and external, each having different surface treatment techniques such as machined, blasted, acid-etched, hydroxyapatite-coated, titanium plasma-sprayed, and oxidized [59]. Therefore, in case of failures, repair and follow-ups can become extremely complicated if dentists do not classify the implant system correctly. Several studies have developed ML models to identify dental implants and their brand names with high accuracy. The study conducted by Sukegawa et al. demonstrated the successful utilization of multi-task DL for developing a classifier that can categorize implant brands using dental panoramic radiographic images [36]. Marginal bone loss (MBL) is a significant contributor to dental implant failure. Zhang et al. employed ML algorithms that utilize the internal architecture of bone tissue to forecast the onset of significant marginal bone loss (MBL) in dental implants. The study used four different ML models, of which SVM produced the most accurate results, with an AUC of 0.967 [37].

Plaque accumulation causes peri-implantitis, an inflammatory disease, which can lead to bone loss around the insertion site of implants. In a cohort study conducted by Mameno et al., an ML-based model was developed to detect the onset of peri-implantitis. Three ML models were used to analyze the risk factors linked with the development of peri-implantitis. The AUC of the random forest model (the most precise model in this study) was 71% [38]. 

In the last decade, there have been incredible innovations and advancements in dental implantology. Despite its relatively recent widespread clinical implementation by dentists, AI has demonstrated potential in assisting clinicians to reduce the risk of failures and minimize the time required for repairs in case of failures.

### 3.6. Applications of AI in the Field of Orthodontics

The objective of orthodontic treatment is to restore individual normal occlusion and enhance facial aesthetics in patients with malocclusion [60]. The field of orthodontics has seen significant investments in research and development of AI technologies, with an estimated economic return of 3.6 billion dollars [9]. Decision-making is an integral part of orthodontics, and AI has opened new horizons in dentistry by providing multiple agile architectures with adequate precision and accuracy. Although the final diagnosis still must be made by dentists, ML can act as a potent auxiliary tool in the process of orthodontic treatment. Recent studies employing ML models in orthodontics focused on areas such as tooth segmentation, landmark identification, treatment planning, growth determination, the need for orthodontic extraction, and impact on face attractiveness. 

Timing plays a vital role in orthodontic planning for growing patients [40]. It has been stated that clinical effects of the therapy are observed at the earliest in patients whose treatment commenced at the right time, i.e., during the peak growth period [61]. Determining the proper time to initiate treatment is based on the patient’s skeletal maturation and bone age [62]. The assessment of skeletal maturity is essential to carry out an effective treatment with the use of orthodontic appliances in children with class II malocclusion [61]. Kök et al. developed an ML model to determine growth by cervical vertebrae maturation stages. Seven AI classification algorithms were used on cephalometric radiographs from 300 children and adolescents aged between 8 and 17. ANN was the most stable algorithm in this study, with an accuracy of 55.6%–93% [40]. A necessary step in devising the optimal orthodontic treatment plan is the recognition of anatomic landmarks in radiographs, with the purpose of measuring various angles, distances, and ratios. Kunz et al. employed a CNN algorithm (using Keras & Google Tensorflow) to analyze cephalometric X-rays with a level of accuracy close to clinicians (considered the current gold standard) [41].

Another integral part of treatment planning is whether to perform extraction and to determine the teeth that should be extracted. Jung et al. collected lateral cephalograms from 156 patients and selected a two-layer neural network for ML. The overall success rate of the decision-making process between extraction and non-extraction was 93% [39].

### 3.7. Applications of AI in the Field of Forensic Dentistry

The process of forensic identification involves comparing the characteristics present in a sample of evidence to those of a known reference sample to draw conclusions about the source of the evidence [63]. Forensic odontology is a reliable method for identification based on robust dental traits [64]. Most AI-based studies in this area focused on person identification, classifying tooth types, and estimation of age using teeth.

Tooth detection and numbering in dental radiographs is one of the initial stages in most forensic odontology tasks. Leite et al. developed an AI-based tool that demonstrated improved speed and accuracy in detecting and segmenting teeth on panoramic radiographs compared to manual segmentation [42].

Nowadays, with the rise in crime, missing people, and natural disasters all over the world, human identification has become increasingly important [65]. Sathya et al. adopted the popular CNN architecture, AlexNet, to conduct human identification through dental radiographs in a two-stage process. In the classification stage, the neural network identifies the tooth number in the post-mortem query image (images taken after the candidate’s death). In the second stage, candidate matching is performed by comparing the query image with corresponding images in the ante-mortem database (images taken when the candidate was alive) [43].

Furthermore, teeth are considered to be useful biological indicators for estimating the age of an individual, as they can remain intact for an extended period of time after death. Farhadian et al. created a neural network age prediction model using the pulp-to-tooth ratio in canines. The model was trained on radiographs of 300 subjects between the ages of 14 and 60 years and was able to predict age with an acceptable mean absolute error (MAE) of 4.12 years [44].

### 3.8. Applications of AI in the Field of Cariology

Tooth decay, commonly known as dental caries, is a widespread and chronic condition that affects a considerable portion of the world’s population [66]. Dental caries is caused by three primary factors, which include microorganisms that produce acid, dietary carbohydrates, and host factors such as eating patterns [67]. Caries that are not treated in permanent teeth are now the most prevalent health issue worldwide, affecting 34.1% of the population, and can lead to various oral diseases [68]. AI models are particularly useful in predicting carious lesions early on to decrease further treatment needs. With the help of ML, caries can be detected and classified on different tooth surfaces.

Detecting dental caries at an early stage is crucial for providing appropriate prevention and treatment to patients with this condition [69]. Various studies utilized different input data types for their ML prediction models. Tamaki et al. developed a dental caries prediction model using data mining techniques. Their CNN model utilized several input variables, including salivary levels of mutans streptococci and lactobacilli, salivary pH, and the frequency of sweet snack consumption, resulting in a sensitivity of 0.73 and specificity of 0.77 [45]. In 2020, a study was carried out in China to predict dental caries in the high-risk population of geriatric citizens. The study involved 1144 elderly patients, and a prediction model using a generalized regression neural network (GRNN) was created to determine the possibility of tooth decay. The results from the GRNN early warning model showed that cases with a history of toothache and tobacco use were more susceptible to dental caries [46].

Wang et al. studied the correlation between dental caries and genetics using logistic regression models. A comprehensive gene set analysis was performed for dental caries, and five gene sets were identified as potential factors for caries development [70].

The diagnosis and classification of carious lesions is also a difficult task. ML can help dentists to improve diagnosis as a reliable second opinion [71]. Neural networks and image processing techniques were used to detect caries on proximal and occlusal surfaces of teeth [71,72]. Moutselos et al. utilized a dataset of 88 dental images for training a DL model (Mask R-CNN) to detect and classify caries specifically on the occlusal surfaces of teeth, where caries is most commonly found inside the pits and fissures. The model attained an F-measure of 0.778 [47].

### 3.9. Applications of AI in the Field of Pediatric Dentistry

Pediatric dentistry contributes greatly to ensuring the proper nutrition and health of children in society by maintaining their ability to chew properly through the preservation of primary teeth [73]. Childhood caries and preadolescent abnormalities are among the most recent areas of interest for ML implementation. Untreated dental plaque on primary teeth can result in a variety of oral diseases, including caries, gingivitis, and periodontitis, which can have negative consequences on the growth and development of permanent dentition [74]. Detecting plaque on primary teeth can be challenging since distinguishing a small amount of plaque from the tooth is difficult. It requires assessment by a clinician, and when dealing with an uncooperative child, it becomes even more challenging. You et al. developed an AI model that successfully identified plaque on primary teeth with satisfactory accuracy using intraoral photos captured by an affordable digital camera, making it feasible for parents to use at home [48].

Park et al. identified risk factors such as sugar consumption and allergic disease for their early childhood caries (ECC) prediction models. Analysis of the data of 4195 children using ML-based models showed a favorable performance in predicting early childhood caries and high-risk groups for ECC [75].

The assessment of children’s oral health by clinicians is crucial for identifying their treatment requirements. However, clinical diagnosis is not universally feasible, and accessibility to it varies. Wang et al. employed machine learning to forecast children’s oral health treatment needs using sociodemographic factors like language, age, sibling count, dental insurance, and parental occupation. This method can be particularly beneficial in areas where children lack access to dental professionals since it is user-friendly for parents and school personnel [49].

The most common abnormality of supernumerary teeth is mesiodens, with a prevalence ranging from 0.15% to 1.9% [76]. Mesiodens can cause multiple complications, such as eruption disorders and crowding. Therefore, early detection of this abnormality in children is crucial for preventing such complications. A study by Ahn et al. utilized panoramic radiographs from 1100 children to classify mesiodens in primary or mixed dentition using DL models. The classification accuracy of ML models was found to be higher than 90% [50]. 

The main goal of most pediatric dentistry procedures is preventive dental care. Therefore, AI can have a great impact on improving oral health among future elderly populations.

### 3.10. Others

Modern dentistry emphasizes the utilization of minimally invasive procedures, leading to a recent increase in the adoption of lasers as a preferred alternative to traditional dental instruments [77]. Engelhardt et al. utilized an ML algorithm to classify reflected light spectra into different tissue types (e.g., bone, mucosa, nerve) in laser dentistry. The main objective was to provide a warning or temporarily halt the use of the laser when critical tissue was at risk of being removed. The model exhibited an average misclassification rate of 0.02, which can be deemed a crucial step toward enhancing the safety of laser surgery in the oral cavity [78]. 

AI-based computer-vision algorithms have become increasingly popular in recent years for automatically detecting and classifying dental restorations in panoramic radiographs. Abdalla-Aslan et al. designed an ML model that can detect different dental restorations in panoramic radiographs automatically. The study examined 738 dental restorations across 83 panoramic images and showed that the algorithm was able to detect 94.6% of the restorations, indicating the excellent performance of the model [51].

Osteoarthritis is characterized as a degenerative condition that can bring about significant changes in joint cartilage tissues, ultimately resulting in severe pain and impaired joint function [79]. Temporomandibular joint osteoarthritis (TMJOA) is an important subtype in the classification of temporomandibular disorders [80]. Choi et al. created an AI model to detect TMJOA on orthopantomograms (OPGs) by using CBCT results that have already been confirmed by experts. The DL model was able to match the sensitivity of an expert while achieving a more optimal trade-off between sensitivity and specificity [52]. A summary of the recent applications for AI can be found in Table 1. 

## 4. Discussion

AI has revolutionized different dental specialties by providing agile and cost-effective models for clinical diagnosis and assessment of numerous oral diseases, promising a better future for healthcare systems around the globe.

The present study aimed to provide a thorough review of recent literature on the application of AI in various dental specialties. Because of its ability to recognize delicate and complex patterns from all sorts of databases (especially dental radiographs), AI can be particularly helpful in clinical dental practice in the near future. For instance, ML can aid in orthodontics by identifying local skeletal patterns from OPGs and lateral cephalograms in specific populations who share a common ancestry or are native to a particular geographic location. By doing so, the need for trial and error can be eliminated, and only the necessary treatments can be considered for that particular population. Another area in which AI shines brightly is cariology [81]. AI algorithms can discern tooth caries from other dental lesions in the oral cavity with acceptable precision. Dental caries is still one of the most widespread chronic diseases globally and left undetected, it can cause numerous oral diseases such as PBL. Therefore, automating caries detection from images by implementing AI-based image processing methods can reduce the financial burden of governmental healthcare sectors. Most importantly, the current study demonstrated recent applications of AI in pediatric dentistry, a very significant subfield that had been neglected in most reviews. The ever-growing costs and complications of dental treatment have made preventive dentistry a highly sought-after approach for authorities worldwide. Thus, by implementing ML methods on the gathered data from pediatric patients and detecting dental defects early on, a decrease in dental treatment costs can be attained for future generations, leading to the possibility of a healthier tomorrow.

Moreover, in recent decades, the World Health Organization (WHO) has emphasized the importance of oral healthcare among children, pregnant women, and elderly populations, but unfortunately, monitoring these target groups with conventional methods cannot be performed effectively in developing countries because of financial issues and the lack of resources. Due to its relatively low operating budget, AI can be particularly helpful in less affluent or sanctioned countries that are in dire need of quality surveillance systems to improve their healthcare systems in a short period of time.

Although the mentioned advantages are significant, there are still complexities associated with AI utilization in dentistry. Problems with data selection and preprocessing, as well as defining gold standards, hampers results in terms of robustness and comparability, especially since dental data availability is limited due to data and protected patient information regulations, resulting in biased datasets. Moreover, specific algorithms are not easily generalized for similar tasks, potentially leading to biased outcomes in some instances. Additionally, the current processing power for AI computations is limited, causing performance drops when the layers of a deep learning model increase or when training on large datasets [82]. Although AI-specific chips are being developed, there is still room for hardware and software improvements [83]. Another major challenge is defining clear ethical guidelines for the clinical use of AI. The advancement of AI has raised ethical issues such as privacy violations, security risks, and transparency. Protecting patient data through strong encryption methods is crucial, but achieving healthcare privacy may be difficult with extensive data sharing. Integrating AI into healthcare may worsen existing health disparities due to potential algorithmic bias [84]. 

To address the current challenges effectively, deeper research is required in key areas, including advanced data techniques for creating unbiased datasets, the development of generalized algorithms, optimization of processing power, and the establishment of clear ethical guidelines for responsible AI integration in dental healthcare.

## 5. Conclusions

Despite AI’s ability to assist clinicians as an auxiliary tool in multiple criteria, results derived from AI models should be interpreted with caution. AI models still face some drawbacks and have not largely entered routine dental practice. Therefore, some key factors should be considered to cope with the current challenges and limitations encountered while implementing ML in clinical dentistry:

- With the help of modern computational processing power, more sympathetic AI models should be devised that can imitate human understanding and emotions to a higher degree for the purpose of building trust with the patients and reassuring them in stressful situations
- Data management should move towards decentralized methods with the help of cloud services in order to enable individual users, who have limited processing power and information, to utilize vast amounts of polished data for training their models locally

Furthermore, systematic reviews should be conducted regarding the use cases of AI in dentistry so as to give researchers a deeper insight into this modern technology. 

## Figures and Tables

**Table 1:** Overview of AI implementation in various dental specialties.

Author	Application	Data Set	AI Architecture	Performance
Danks et al. [15]	PBL measurement	340 images	CNN	Percent of correct key points (PCK): 0.83
Kim et al. [22]	Prediction of chronic periodontitis severity	692 mouthwash samples	NN RF SVM RLR	Accuracy: 0.93 AUC: 0.96 Sensitivity: 0.96 Specificity: 0.81
Lee et al. [11]	Diagnosis for periodontal disease	45,553 participants	DT	Accuracy: 0.85
Shimpi et al. [23]	Estimation of periodontitis	11,048 patients	DT	Specificity: 0.90
Gao et al. [14]	Estimation of pain after RCT	300 patients	ANN	Accuracy: 0.95
Saghiri et al. [24]	Locating minor apical foramen	50 images	ANN	Accuracy: 0.96
Fukuda et al. [25]	Vertical root fracture detection	300 images	CNN	Precision: 0.93 F measure: 0.83 Recall: 0.75
Benyo et al. [26]	Detecting medial axis of the root	Micro CT cross-section images	ANN	Accuracy: 0.95
Sukegawa et al. [27]	Classification of third molar	1330 images	CNN	Accuracy: 0.84
Vranckx et al. [28]	Molar angulation measurement	838 images	CNN	Accuracy: 0.80–0.98
Hiraiwa et al. [29]	Root morphology measurement	760 image sets	CNN	Sensitivity: 0.85–0.87
Patcas et al. [30]	Facial attractiveness measurement	146 patients	CNN	Mean difference: 1.22
Kuwada et al. [31]	Cleft detection	383 images	CNN	Accuracy: 0.82
Alzubaidi et al. [32]	Osteoporosis classification	575 images	SVM	Accuracy: 0.92
Kwon et al. [33]	Diagnosis for cysts and tumors of both jaws	1282 images	CNN	Accuracy: 0.956 AUC: 0.94 Sensitivity: 0.889 Specificity: 0.956
Takahashi et al. [34]	Classification of partially edentulous arches	1184 images	CNN	Accuracy: 0.995 Maxilla 0.997 Mandible
Takahashi et al. [35]	Identification of prostheses	1904 images	CNN	Precision: 0.59–0.93
Sukegawa et al. [36]	Classification of dental implant brand	9767 images	CNN	Accuracy: 0.9908
Zhang et al. [37]	Detect marginal bone loss	81 patients	SVM	AUC: 0.967
Mameno et al. [38]	Predicting peri-implantitis	489 patients	SVM	AUC: 0.64
Jung et al. [39]	Diagnosis of tooth extractions for orthodontic treatment	Lateral Cephalograms, 156 subjects	Neural network	Accuracy: 0.93
Kök et al. [40]	Growth determination	300 images	ANN	Accuracy: 0.78–0.93
Kunz et al. [41]	Cephalometric analysis	1792 images	CNN	Absolute mean difference: 0.42–2.18
Leite et al. [42]	Tooth segmentation	153 images	CNN	Precision: 0.96
Sathya et al. [43]	Human identification	3159 images	CNN	Accuracy: 0.95
Farhadian et al. [44]	Age estimation	CBCT scans, 300 subjects	Neural network	MAF: 4.12 Years
Tamaki et al. [45]	Dental caries prediction	Saliva samples, 500 subjects	CNN	Sensitivity: 0.73 Specificity: 0.77
Liu et al. [46]	Dental caries prediction	1144 subjects	GRNN	AUC: 0.626
Moutselos et al. [47]	Caries classification	88 images	CNN	Accuracy: 0.67–0.89
You et al. [48]	Plaque detection	984 images	CNN	MIoU: 0.726 ± 0.165
Wang et al. [49]	Evaluating children’s oral condition	545 subjects	XGboost	Sensitivity: 0.93
Ahn et al. [50]	Mesiodens classification	1100 images	CNN	Accuracy: 0.927
Abdalla-Aslan et al. [51]	Classification of dental restorations	83 images	SVM	Accuracy: 0.93
Choi et al. [52]	Detection of TMJOA	1189 images	CNN	Accuracy: 0.78

## Data Availability

The corresponding author can provide data upon a reasonable request.
